# BMI-for-age *Z*-score trajectories in early childhood: a comparison of GMM and KML and associated antenatal and postnatal factors

**DOI:** 10.1007/s12519-026-01057-y

**Published:** 2026-07-23

**Authors:** Ting Zhang, Zi-Yun Li, Zhen-Yu Zhang, Mai Gao, Li Zhang, Hui-Juan Liu, Hai-Jun Wang, Yan Li

**Affiliations:** 1 Clinical Research Center for Children’s Health and Diseases, Shandong Provincial Maternal and Child Health Care Hospital Affiliated to Qingdao University, Jinan, China; 2 School of Clinical Medicine, Shandong Second Medical University, Weifang, China; 3 Department of Maternal and Child Health, School of Public Health, National Health Commission Key Laboratory of Reproductive Health, Peking University, Beijing, China

**Keywords:** BAZ trajectory, Children, Growth mixture modeling, K-means for longitudinal data, Possible risk of being overweight, Pediatric

## Abstract

**Background:**

Early-life growth trajectories, especially during the first two years, are important for future health. However, in the context of rapidly rising childhood obesity, it is essential to characterize BMI-for-age *Z*-score (BAZ) trajectories in early childhood, compare optimal modeling approaches, and identify associated risk factors.

**Methods:**

This prospective birth cohort study enrolled participants in Jinan, Shandong from 2019 to 2022. World Health Organization (WHO)-standardized BAZ scores were calculated for children at eight timepoints (0–24 months). Sex-specific BAZ trajectories were derived using growth mixture modeling (GMM) and k-means for longitudinal data (KML). The possible risk of being overweight (BAZ > 1) at age three years was predicted using logistic regression with five folds cross-validation, comparing model performance via the area under the curve (AUC). Associations between trajectory classification and antenatal and postnatal factors were examined.

**Results:**

A total of 4629 mother-infant dyads were enrolled in this study. The KML-derived four-class trajectory model demonstrated optimal predictive performance for possible risk of being overweight at age three years, achieving an AUC of 0.749 and significantly outperforming the GMM model (AUC = 0.571). Male sex [odds ratio (OR) = 1.314; 95% confidence interval (95% CI): 1.098, 1.572], high birth weight (OR = 4.739; 95% CI: 3.594, 6.250), multiparity (OR = 1.265; 95% CI: 1.003, 1.594), and gestational hypertension (OR = 2.581; 95% CI: 1.387, 4.801) were identified as risk factors for high growth trajectory.

**Conclusions:**

The KML model was identified as the more effective approach for delineating BAZ trajectories and predicting the risk of being overweight in our study population. Furthermore, male sex, high birth weight, multiparity, and gestational hypertension were identified as key antenatal and postnatal factors associated with higher growth trajectories.

**Graphical Abstract:**

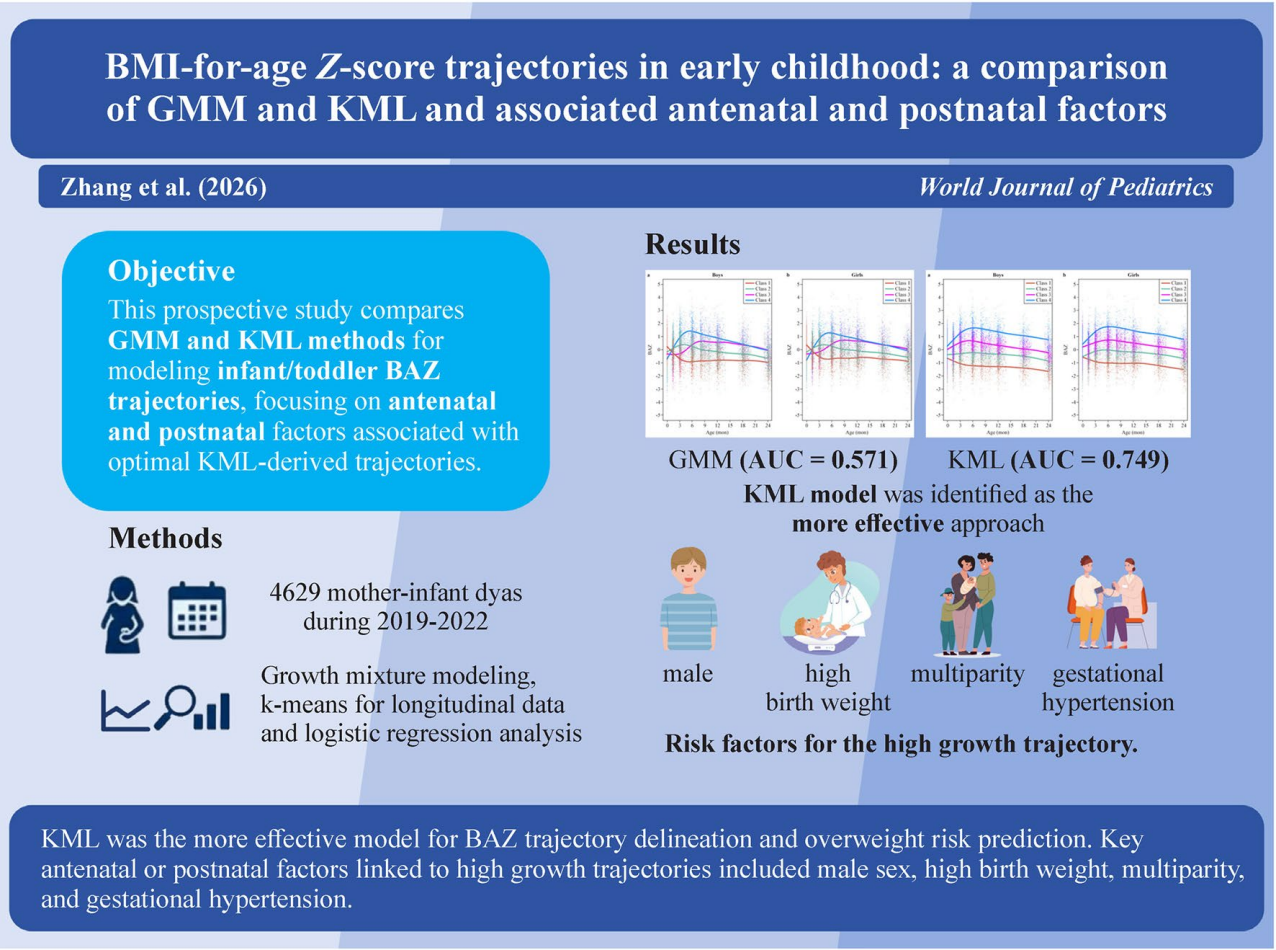

**Supplementary Information:**

The online version contains supplementary material available at 10.1007/s12519-026-01057-y.

## Introduction

Early life, particularly during the first two years, constitutes a critical window for long-term metabolic health [1]. A growing body of evidence underscores the profound impact of body mass index (BMI) dynamics during infancy and early childhood on the subsequent risk of obesity and cardiometabolic diseases in adulthood [2, 3]. Compared to single-time-point measurements, characterizing developmental BMI trajectories provides a more robust and clinically meaningful approach for monitoring body size evolution, predicting later health outcomes, and identifying opportunities for early intervention. There are now 35.5 million overweight children under five years of age globally; this is an increase of 2.4 million since 2000. In Eastern Asia, the proportion of overweight children under five increased significantly from 6.5% in 2012 to 10.1% in 2024 [4]. Current data in children under six years old in China reveals a prevalence of being overweight and obesity of 9.7%. Even with a reduction from levels in 2020, this rate represents a substantial and pressing public health concern [5].

Growth mixture modeling (GMM) [6–10] and k-means for longitudinal data (KML) [11, 12] are two commonly used methods to achieve this goal. However, recent methodological literature highlights a critical challenge: growth pattern detection is highly dependent on the statistical method used. For instance, Massara et al. [13] demonstrated that different clustering configurations (e.g., latent class mixed models compared with k-means) applied to the same cohort yield substantially different trajectory patterns and group assignments, with agreement rates sometimes as low as 58%. Similarly, Lu et al. [14] noted that model-based and algorithm-based approaches make different underlying assumptions about data distribution and variance, further complicating cross-study comparability. This methodological heterogeneity has also been reported in systematic reviews, emphasizing the difficulty in comparing childhood BMI trajectories across global cohorts [15]. Whilst longitudinal growth patterns from birth through early childhood have been increasingly studied globally, including in non-Western and low-to-middle-income countries (LMICs) [16], a significant gap in the literature remains. Most existing studies pre-select a single trajectory modeling approach without validating its clinical utility, potentially leading to biased identification of risk factors.

Many studies that have prospectively explored BMI trajectories have focused on childhood and adolescence [17–22]. Relatively few have studied trajectories from birth to early childhood [10, 23]. The first two years of life in children have been identified as a sensitive window for the later development of obesity and cardiometabolic diseases [24, 25]. Identifying groups of young children who are on trajectories associated with a high risk of developing obesity may facilitate targeted early intervention. For example, a Finland birth cohort study identified three distinct clusters of BMI trajectories in children which were found to be associated with deviations from typical growth patterns and long-term metabolic risk [2]. Similarly, longitudinal analyses showed that rapid increases in BMI during infancy were associated with higher fat mass and insulin resistance in later childhood [3, 26]. There are two objectives of this prospective study: (1) evaluating method dependence of BMI-for-age *Z*-score (BAZ) trajectory detection (GMM compared to KML) and determine which method better predicts the risk of being overweight at age of three years; (2) using the optimally defined trajectories to describe the impact of critical antenatal and postnatal factors on early childhood growth.

## Methods

This prospective cohort study utilized data from the Maternal and Child Birth Cohort of the Shandong Provincial Maternal and Child Health Care Hospital. Data use was approved by the Department of Obstetrics, the Department of Child Health Care, the Child Health Research Center, and the ethics review committee of the hospital (No. 2025–040). Written informed consent was obtained from the parents of all children in this birth cohort.

### Study participants

Children included in this study were born between January 2020 and August 2022 and were regularly followed up in the Department of Child Health Care at Shandong Provincial Maternal and Child Health Care Hospital. Inclusion criteria were: (1) gestational age (GA) at birth ≥ 37 weeks and < 42 weeks; (2) singleton with no birth defects; (3) followed up on at least three occasions from one month to two years of age (at least one recording at 1, 3, and 6 months of age, at least one recording at 9, 12, and 18 months of age, and one recording at 24 months of age). Exclusion criteria were: (1) presence of genetic and metabolic diseases affecting growth and development; (2) children with missing birth weight or GA information.

### Anthropometric measurements of infants and toddlers

Length (cm) and weight (kg) were measured supine using an integrated electronic length and weight scale (KANGWA WS-RTG-1G, Suzhou, China), which has a length range of 30–105 cm (digital counter accuracy: 1 mm) and a weight range of 0–60 kg (digital counter accuracy: 0.05 kg). Two child health care nurses independently measured both length and weight; the average value was recorded and uploaded to the child health examination system. Acceptable inter-observer technical error of measurement (TEM): length ≤ 7 mm, weight ≤ 100 g.

BMI was defined as weight/length square (kg/m^2^). Prior to trajectory modeling, rigorous data preprocessing and cleaning were conducted. We excluded records with negative age values as invalid data and coded unrecorded weight or length measurements as missing. The BAZ of children at each time point were calculated according to the World Health Organization (WHO) standard, and outliers with an absolute value of BAZ greater than five were excluded [27]. Subsequent trajectory modeling was performed on the cleaned and standardized dataset. At the same time and according to the WHO definition [28], for children under five years old, if the BAZ of an individual is greater than two at a certain time point, the individual is judged to be overweight/obese at that time point. If the BAZ is greater than one at a certain time point, the individual was judged to be at possible risk of being overweight/obese at that time point.

### Data collection and variable definitions

Neonatal characteristics, including sex, GA, birth weight, and mode of delivery were recorded on the system by trained midwives. Maternal characteristics, including age at delivery, parity, threatened abortion, gestational infection, gestational diabetes, gestational hypertension, and thyroid dysfunction in pregnancy, were recorded by professional obstetricians. Characteristics above were included as covariates in this study. Early term infants were defined as 37^+0^–38^+6^ weeks GA, and term infants were defined as 39^+0^–41^+6^ weeks [29]. Birth weight was categorized into quartiles based on its distribution in the study population. Advanced maternal age is defined as being 35 or older at the time of delivery [30].

### Statistical analysis

All statistical analyses were performed using R software (version 4.3.2). Two statistical methods, GMM and KML, were used to identify optimal growth patterns in infants and toddlers. GMM is an extension of group-based trajectory modeling (GBTM), which is suitable for parameter modeling of longitudinal data. GMM estimates the average growth curve for each latent class, but also allows for differences between individuals within the same class [31, 32]. The optimal number of latent trajectory groups was determined by balancing statistical fit, model parsimony, and clinical interpretability. Furthermore, we conducted probability-weighted logistic regression analyses for the predictive performance of the GMM model. KML analysis was conducted separately by sex using the “kml” package in R [11]. KML was favored as it does not require normality assumptions, is robust in data convergence, and makes no a priori assumptions about trajectory shape. Detailed descriptions of the GMM and KML procedures, including model specifications, parameter settings, and model evaluation criteria, are provided in the Supplementary Material.

Linear regression models were used to analyze the association between BAZ trajectories in infants and toddlers determined by different methods and children's BAZ at three years of age. The reporting of our predictive modeling adheres to the transparent reporting of a multivariable prediction model for individual prognosis or diagnosis (TRIPOD) guidelines. A logistic regression model was utilized to evaluate the predictive performance of the derived 0–2 years BAZ trajectory classes on the outcome of possible risk of being overweight (BAZ > 1) at age three years. To assess model performance and prevent overfitting, internal validation was conducted using five-fold cross-validation. The dataset was randomly partitioned into five equal folds, iteratively using four folds for training and one-fold for testing. Model discrimination was evaluated by calculating the AUC. The cross-validated mean AUC, along with its 95% confidence interval (95% CI) and standard deviations, was reported to compare the predictive validity of the GMM and KML trajectory groupings.

Logistic regression analysis was used to examine the effects of prenatal and postnatal exposures on the growth trajectories of children. For these analyses, the “moderately low trajectory group” and “moderately high trajectory group” were combined to serve as the reference category, representing clinically normal BAZ development within the – 1 to + 1 standard deviation range. We then investigated factors associated with the “low trajectory group” and the “high trajectory group” by comparing them against this combined normal reference. For logistic regression analyses, covariates were selected through a two-stage process to ensure clinical relevance and mitigate the risk of overfitting. First, a comprehensive set of prenatal and postnatal factors were initially considered based on their established associations with early childhood growth and risk of being overweight in existing epidemiological literature. Second, each of these candidate exposures was evaluated in separate univariate logistic regression models. Only those exposures demonstrating statistical significance (*P* < 0.05) in this initial screening were then included in the subsequent multiple logistic regression models (adjusted model 1, model 2, and model 3). This approach allowed understanding of the combined role of influential prenatal and postnatal factors in growth trajectory differentiation while maintaining model parsimony. The stability of our main findings across different adjustment levels (model 1, 2, and 3) further supports the robustness of our predictor selection.

## Results

### Participant characteristics

**Fig. 1:**
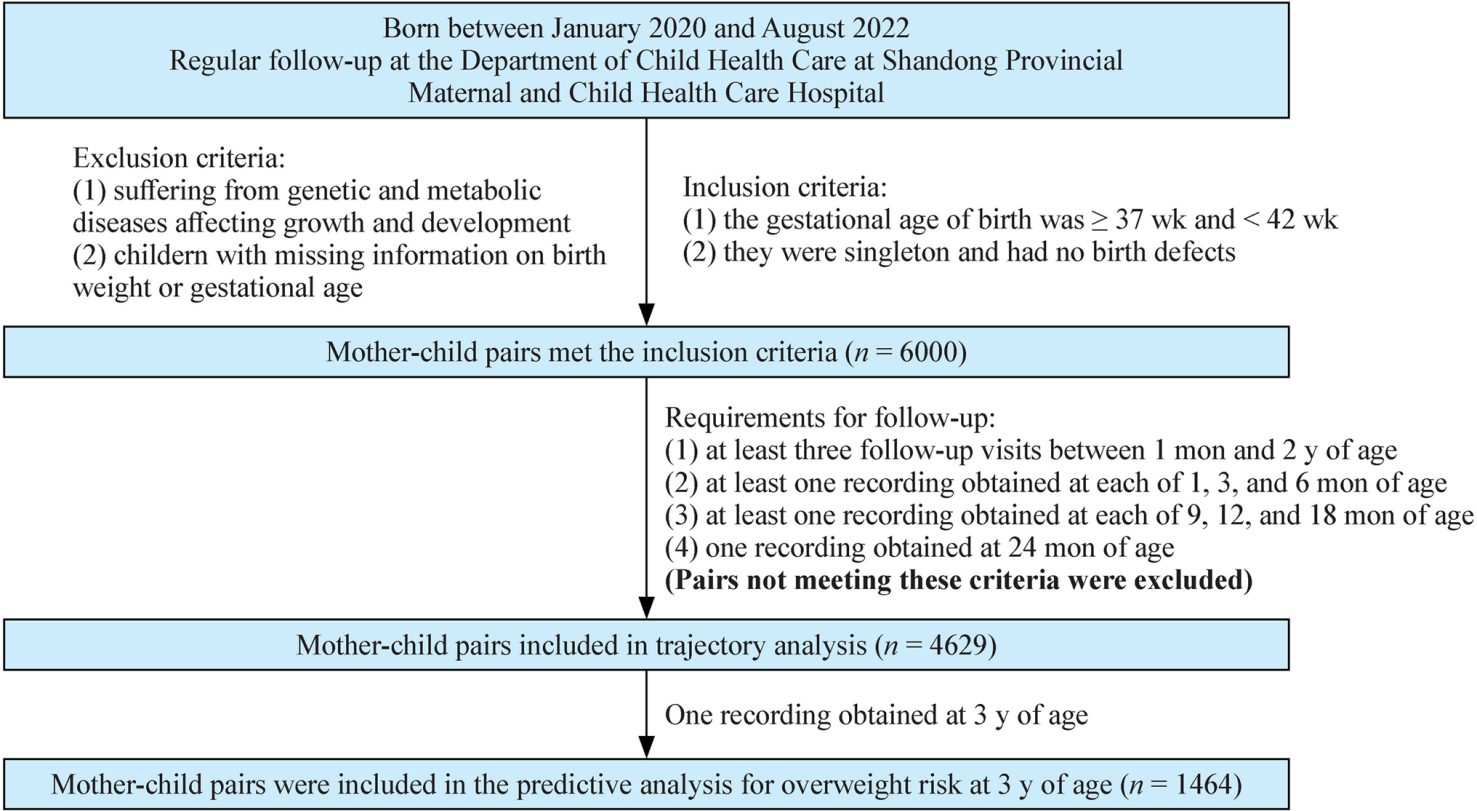
Flowchart of participant inclusion and data collection. *y* year; *wk* week; *mon* month

The flowchart of participant screening is showed in Fig. 1. A total of 4629 mother-child pairs were included, and the basic characteristics are shown in Table 1. The average GA at birth was 39.08 weeks, and the proportion of early term infants was 27.03%. Average birth weight was 3.35 kg. Exclusive breastfeeding accounted for 85.40% of participants at one month of age. Regarding maternal characteristics, 7.39% were of advanced maternal age and 84.75% were primiparous. Furthermore, during pregnancy, 1.34% had threatened abortion, 1.02% had an infection, 1.30% had gestational hypertension, 2.72% had gestational diabetes, and 2.33% had thyroid dysfunction.

**Table 1:** Maternal and child birth cohort characteristics (*N* = 4629)

Characteristics	Results
Infants	
Sex (boy)	2438 (52.7)
Mode of delivery (vaginal)	2964 (64.0)
GA, wk	39.08 ± 0.99
Categorized by GA^a^	
Early-term	1251 (27.03)
Full-term	3378 (72.97)
Birth weight, kg	3.35 ± 0.40
Categorized by birth weight^b^	
Q1	1164 (25.15)
Q2	1176 (25.41)
Q3	1153 (24.91)
Q4	1136 (24.54)
Feeding pattern within 1 mon (breastfeeding)	3953 (85.40)
Mothers	
Advanced maternal age (yes)	342 (7.39)
Parity (primigravida)	3923 (84.75)
Threatened miscarriage (yes)	62 (1.34)
Infections in pregnancy (yes)	47 (1.02)
Gestational hypertension (yes)	60 (1.30)
Gestational diabetes mellitus (yes)	126 (2.72)
Thyroid dysfunction in pregnancy (yes)	108 (2.33)
Offspring in 3 y	
Weight, kg	14.74 ± 1.99
WAZ	0.17 ± 1.00
Height, cm	98.41 ± 3.65
HAZ	0.55 ± 0.95
BMI	15.18 ± 1.39
BAZ	− 0.31 ± 1.08
Possible risk of overweight (yes)	157 (10.72)
Overweight (yes)	39 (2.66)
Obesity (yes)	9 (0.61)

Data was presented as *n* (%) or mean ± standard deviation (SD). ^a^ infants with GA of 37–38 weeks were categorized in early-term, those with GA of 39–41 weeks were categorized in full-term. ^b^birth weight was categorized in quartile, Q1 represented those with birth weight of less than 3.08 kg, Q2 represented 3.08–3.34 kg, Q3 represented 3.34–3.60 kg, and Q4 represented greater than 3.60 kg. *GA* gestational age, *WAZ* weight-for-age *Z*-score, *HAZ* height-for-age *Z*-score, *BMI* body mass index, *BAZ* BMI-for-age *Z*-score

### BMI-for-age *Z*-score growth trajectory

Physical examination data at 0, 1, 3, 6, 9, 12, 18 and 24 months old were used to analyze the trajectory of BAZ from birth to two years; the average number of physical examinations was 6.72 ± 1.18. The distribution of children's physical examination data included in the trajectory analysis is shown in Supplementary Table 1.

**Fig. 2:**
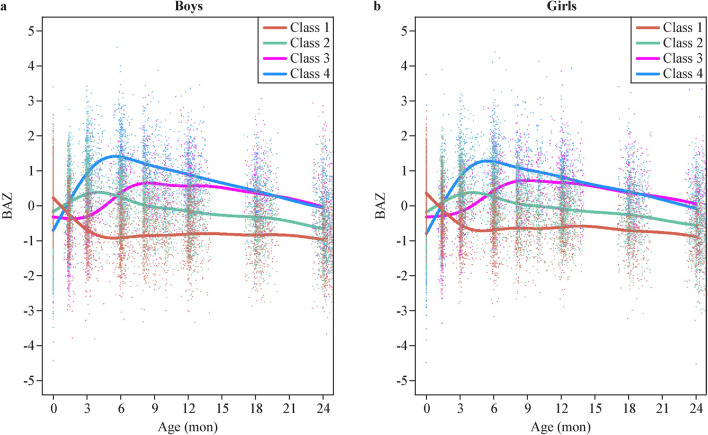
BMI-for-age *Z*-score (BAZ) trajectories in children identified by the four-class growth mixture modeling (GMM) method. **a** results in boys; **b** results in girls. *BAZ* BMI-for-age *Z*-score; *mon* month

Based on the GMM method, four distinct trajectory groups were identified as optimal. The trajectories were categorized as follows: class 1 as the “low trajectory group” (*n* = 1057, 22.83%), class 2 as the “moderately low trajectory group” (*n* = 2063, 44.57%), class 3 as the “moderately high trajectory group” (*n* = 755, 16.31%), and class 4 as the “high trajectory group” (*n* = 754, 16.29%). Classification results are presented in Table 2, and the corresponding growth trajectories are shown in Fig. 2. Detailed BAZ values determined using the GMM method at each time point for the four trajectory groups from birth to two years of age, are shown in Supplementary Table 2. Figure 2 presents the sex-specific distribution and trajectory characteristics of BAZ for the four-category groupings determined by the GMM method. This shows generally consistent trends across sexes: (1) class 1 exhibited a decline before three months that stabilized thereafter without significant catch-up growth; (2) class 2 exhibited catch-up growth before three months of age. Although a declining trend was observed thereafter, the BAZ value remained within the normal range; (3) class 3 was stable before three months, showed pronounced catch-up growth from three to nine months, and subsequently declined while remaining within normal range; (4) class 4 displayed low initial BAZ that rose rapidly above the possible risk of being overweight cutoff (BAZ > 1) within the first six months.

**Table 2:** Group assignments and model parameters of GMM-fitted trajectories

Number of classes	Parameters	AIC	BIC	Entropy	Classes	Proportion	*N*	APPA
2	19	69919.88	70042.25	0.675	class1	53.34%	2469	0.9065
					class2	46.66%	2160	0.8986
3	27	68984.32	69158.20	0.685	class1	22.64%	1048	0.8578
					class2	60.68%	2809	0.8524
					class3	16.68%	772	0.8500
**4**	**35**	**68275.68**	**68501.08**	**0.647**	**class1**	**22.83%**	**1057**	**0.8547**
					**class2**	**44.57%**	**2063**	**0.7772**
					**class3**	**16.31%**	**755**	**0.7483**
					**class4**	**16.29%**	**754**	**0.8335**
5	43	68046.56	68323.48	0.622	class1	12.72%	589	0.7910
					class2	40.01%	1852	0.7607
					class3	15.54%	673	0.7402
					class4	20.70%	958	0.6740
					class5	12.03%	557	0.8166

The bold entries highlight the selected optimal model (three classes), determined by minimizing BIC, maximizing entropy, and ensuring APPA > 70% for each group and a minimum group proportion > 5%. *GMM* growth mixture modeling, *AIC* Akaike information criterion, *BIC* Bayesian information criterion, *APPA* average of posterior probability of assignment

Optimal classification determined by the KML method was a binary classification (Supplementary Fig. 1). While a two-class solution was identified as optimal by KML, we also explored other solutions, including a three-class model, for subsequent analysis. For comparison with the growth trajectories identified by the GMM method, four-class classification was also selected. Consequently, both the two-class and three-class models (derived from KML analysis) were included in the subsequent analysis of their association with possible risk of being overweight at three years of age. Optimal classification was determined based on statistical criteria combined with clinical relevance. The BAZ values at different time points for the dichotomous and trichotomous trajectories from birth to two years of age are provided in Supplementary Tables 3 and 4, respectively, with the corresponding trajectory plots shown in Supplementary Fig. 2.

**Fig. 3:**
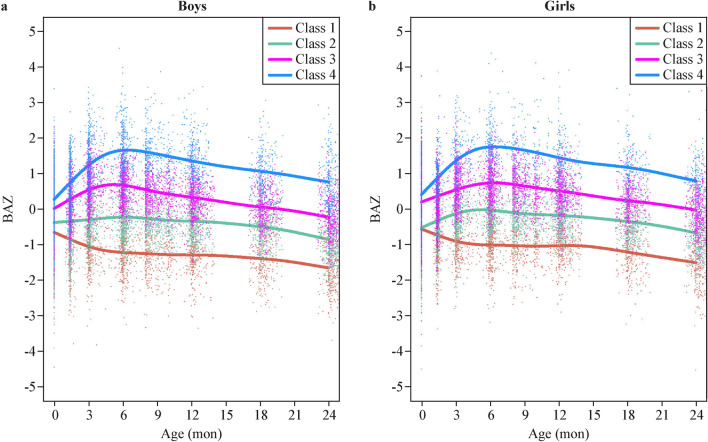
BMI-for-age *Z*-score (BAZ) trajectories in children identified by the four-class k-means for longitudinal data (KML) method. **a** results in boys; **b** results in girls. *BAZ* BMI-for-age *Z*-score; *mon* month

The four-category solution determined by the KML method was labeled as follows: (1) class 1 as the “low trajectory group” (*n* = 886, 19.14%); (2) class 2 as the “moderately low trajectory group” (*n* = 1598, 34.52%); (3) class 3 as the “moderately high trajectory group” (*n* = 1515, 32.73%); (4) class 4 as the “high trajectory group” (*n* = 630, 13.61%). The BAZ at different time points for these specific groups and the four-category BAZ trajectories identified using the KML method are presented in Supplementary Table 5, with the corresponding growth trajectories illustrated in Fig. 3. Figure 3 presents the sex-specific distribution and trajectory characteristics of BAZ in the four-category grouping determined using the KML method. This showed generally consistent trends across sexes: (1) class 1 exhibited a consistent decline; (2) class 2 demonstrated a slow catch-up growth before six months, followed by a declining trend thereafter while remaining within the normal range; (3) class 3 remained within the normal range, showing an initial increase followed by a decrease, with six months of age as the turning point; (4) class 4 had a BAZ slightly higher at birth than class 3, which rose rapidly above the possible risk of being overweight cutoff (BAZ > 1) within the first six months and subsequently declined, yet remained above this cutoff.

### BMI-for-age *Z*-score trajectories as a predictor of possible risk of being overweight at three years of age

A total of 1464 children had BAZ measurements available at three years of age. Of these, 157 children (10.72%) were classified as being at possible risk of being overweight (BAZ > 1) at three years of age. In the analysis utilizing the four-class trajectory determined using the GMM method and after adjusting for sex, mode of delivery, GA, birth weight, and exclusive breastfeeding for the first month (adjusted model 1), the BAZ of children in class 4 at age three was significantly higher than that of their peers in class 1, class 2, and class 3 by 0.251 (95% CI: 0.187, 0.314), 0.220 (95% CI: 0.142, 0.298), and 0.085 (95% CI: – 0.113, 0.282), respectively. Furthermore, the BAZ in class 4 was 0.433 (95% CI: 0.280, 0.585); this was higher than in the combined class 1–3 group.

Similarly, in the KML-defined four-category trajectory, the BAZ of class 4 children at age three exceeded that of classes 1–3 by 0.689 (95% CI: 0.627, 0.751), 0.682 (95% CI: 0.605, 0.759), and 0.788 (95% CI: 0.629, 0.947), respectively. The difference was even more pronounced when compared with the combined low-trajectory groups (class 1–3), with a BAZ increase of 1.275 (95% CI: 1.121, 1.428). Associations between class 4 membership and higher BAZ at three years were strongest in the trajectories determined using the KML method. These findings remained consistent after further adjustments for maternal factors (adjusted model 2) and a comprehensive adjustment including all covariates from both models (adjusted model 3). The detailed results are presented in Table 3.

**Table 3:** Association of different early childhood BAZ groups with BAZ at 3 y

Items	Crude model (*N* = 1464)	Adjusted model 1 (*N* = 1461)	Adjusted model 2 (*N* = 1461)	Adjusted model 3 (*N* = 1461)
GMM (four-category trajectories)				
class 4 vs. class 1	0.221 (0.162, 0.281)	0.251 (0.187, 0.314)	0.229 (0.170, 0.289)	0.259 (0.195, 0.323)
class 4 vs. class 2	0.194 (0.118, 0.271)	0.220 (0.142, 0.298)	0.194 (0.116, 0.271)	0.219 (0.141, 0.297)
class 4 vs. class 3	0.031 (– 0.161, 0.224)	0.085 (– 0.113, 0.282)	0.049 (– 0.139, 0.236)	0.097 (– 0.096, 0.289)
class 4 vs. class 3 + class 2 + class 1	0.392 (0.243, 0.542)	0.433 (0.280, 0.585)	0.404 (0.254, 0.554)	0.440 (0.287, 0.593)
KML (two-category trajectories)				
class 2 vs. class 1	1.045 (0.948, 1.141)	1.054 (0.957, 1.151)	1.053 (0.956, 1.150)	1.052 (0.955, 1.150)
KML (three-category trajectories)				
class 3 vs. class 1	0.801 (0.735, 0.868)	0.805 (0.738, 0.872)	0.803 (0.737, 0.870)	0.803 (0.735, 0.870)
class 3 vs. class 2	0.808 (0.691, 0.924)	0.803 (0.686, 0.920)	0.799 (0.682, 0.916)	0.796 (0.678, 0.913)
class 3 vs. class 2 + class 1	1.094 (0.980, 1.207)	1.094 (0.980, 1.208)	1.100 (0.986, 1.214)	1.091 (0.977, 1.206)
KML (four-category trajectories)				
class 4 vs. class 1	0.696 (0.636, 0.755)	0.689 (0.627, 0.751)	0.697 (0.637, 0.757)	0.691 (0.629, 0.753)
class 4 vs. class 2	0.685 (0.609, 0.761)	0.682 (0.605, 0.759)	0.682 (0.606, 0.759)	0.681 (0.603, 0.758)
class 4 vs. class 3	0.807 (0.651, 0.964)	0.788 (0.629, 0.947)	0.807 (0.650, 0.963)	0.792 (0.633, 0.951)
class 4 vs. class 3 + class 2 + class 1	1.294 (1.141, 1.446)	1.275 (1.121, 1.428)	1.294 (1.141, 1.447)	1.275 (1.121, 1.429)

Data was presented as regression coefficient (95% confidence interval, 95% CI). Crude model was not adjusted for any variable. Adjusted model 1 was adjusted for infant sex, mode of delivery, gestational age, birth weight, feeding patterns within 1 mon (breastfeeding). Adjusted model 2 was adjusted for parity age, parity, threatened miscarriage, infections in pregnancy, gestational hypertension, gestational diabetes mellitus; thyroid dysfunction in pregnancy. Adjusted model 3 is adjusted model 1 combined with adjusted model 2. *BAZ* BMI-for-Age Z-score, *GMM* growth mixture modeling, *KML* k-means for longitudinal data, vs. versus

A logistic regression model was conducted to predict the possible risk of being overweight (BAZ > 1) at three years of age. The predictive performance of different BAZ trajectory groups, determined by various methods, was compared, as detailed in Table 4. After adjusting for covariates (sex, mode of delivery, GA at birth, birth weight, and exclusive breastfeeding for the first month; adjusted model 1), models utilizing the four-class GMM, two-class KML, three-class KML, and four-class KML groupings yielded mean five-fold cross-validated AUCs of 0.562 ± 0.055, 0.717 ± 0.022, 0.736 ± 0.029, and 0.748 ± 0.016, respectively. The four-category BAZ trajectory from 0 to 2 years of age, determined using the KML method, demonstrated the best predictive performance for the risk of being overweight at three years. These findings remained largely consistent after further adjustments in model 2 and model 3. To provide a more comprehensive assessment of the performance of the GMM model, especially considering class assignment uncertainty, we further conducted probability-weighted analyses. For the GMM four-class model, these weighted analyses yielded the following AUCs for predicting the risk of being overweight at three years: 0.500 (95% CI: 0.477, 0.523) for the crude model; 0.596 (95% CI: 0.573, 0.619) for adjusted model 1; 0.529 (95% CI: 0.505, 0.553) for adjusted model 2; and 0.607 (95% CI: 0.584, 0.630) for the combined adjusted model 1 and model 2. Of note, these weighted AUC values, even at their highest, remained considerably lower than the performance of the KML models.

**Table 4:** Prediction performance of different early childhood BAZ trajectory groups on possible risk of being overweight at 3 y

Five-fold CV AUC	Crude model (*N* = 1464)	Adjusted model 1 (*N* = 1461)	Adjusted model 2 (*N* = 1461)	Adjusted model 3 (*N* = 1461)
GMM (four-category trajectories)	0.596 ± 0.022	0.562 ± 0.055	0.577 ± 0.073	0.571 ± 0.018
KML (two-category trajectories)	0.710 ± 0.029	0.717 ± 0.022	0.721 ± 0.026	0.697 ± 0.019
KML (three-category trajectories)	0.736 ± 0.025	0.736 ± 0.029	0.743 ± 0.034	0.721 ± 0.020
KML (four-category trajectories)	0.756 ± 0.030	0.748 ± 0.016	0.753 ± 0.024	0.749 ± 0.017

Binary logistic regression was used to predict overweight risk at 3 y. Data was presented as mean ± standard deviations (SD). Means and SD of the area under the receiver operating characteristics curve were reported as the performance metric of each models using five fold cross-validation. Crude model was not adjusted for any variables; adjusted model 1 was adjusted for infant sex, mode of delivery, gestational age, birth weight, feeding patterns within 1 mon (breastfeeding); adjusted model 2 was adjusted for parity age, parity, threatened miscarriage, infections in pregnancy, gestational hypertension, gestational diabetes mellitus; thyroid dysfunction in pregnancy; adjusted model 3 is adjusted model 1 combined with adjusted model 2. *BAZ* BMI-for-age Z-score, *CV* cross-validation, *AUC* area under the curve, *GMM* growth mixture modeling, *KML* k-means for longitudinal data

### Analysis of the influence of prenatal and postnatal factors on BMI-for-age *Z*-score trajectory

Based on statistical performance and practical clinical utility within the context of our study, the four-class KML model was selected as it demonstrated superior predictive performance. Using the moderately low and moderately high trajectory groups as the reference, univariate logistic regression analysis revealed that male sex, high birth weight, multiparity, gestational hypertension and cesarean section were significantly associated with an increased risk of the high trajectory (Table 5). Multiple regression analysis indicated that male sex, high birth weight, multiparity and gestational hypertension remained significant risk factors for the high trajectory (Table 6).

**Table 5:** Association of prenatal and postnatal factors with infant BAZ trajectories

Variables	Low trajectory		High trajectory	
	OR (95% CI)	*P* value	OR (95% CI)	*P* value
Prenatal factors				
Advanced maternal age				
Yes	1.157 (0.875, 1.529)	0.306	1.220 (0.892, 1.668)	0.214
No	1.000		1.000	
Parity				
Multipara	1.016 (0.822, 1.257)	0.882	1.331 (1.061, 1.670)	0.013
Primigravida	1.000		1.000	
Threatened miscarriage				
Yes	0.698 (0.340, 1.433)	0.327	0.875 (0.410, 1.865)	0.729
No	1.000		1.000	
Infections in pregnancy				
Yes	0.903 (0.414, 1.972)	0.799	1.276 (0.584, 2.789)	0.542
No	1.000		1.000	
Gestational hypertension				
Yes	0.777 (0.360, 1.677)	0.520	2.222 (1.225, 4.030)	0.009
No	1.000		1.000	
Gestational diabetes mellitus				
Yes	0.992 (0.627, 1.570)	0.972	0.988 (0.583, 1.675)	0.964
No	1.000		1.000	
Thyroid dysfunction in pregnancy				
Yes	1.126 (0.700, 1.811)	0.626	0.890 (0.490, 1.617)	0.702
No	1.000		1.000	
Postnatal factors				
Categorized by GA^a^				
Early-term	1.105 (0.935, 1.305)	0.242	1.159 (0.959, 1.401)	0.126
Full-term	1.000		1.000	
Birth weight categories^b^				
Q1	1.459 (1.204, 1.768)	< 0.001	0.608 (0.442, 0.835)	0.002
Q2	1.000		1.000	
Q3	0.821 (0.665, 1.014)	0.067	1.432 (1.107, 1.853)	0.006
Q4	0.477 (0.372, 0.611)	< 0.001	2.701 (2.130, 3.425)	< 0.001
Sex				
Boys	0.902 (0.777, 1.047)	0.174	1.456 (1.222, 1.734)	< 0.001
Girls	1.000		1.000	
Mode of delivery				
Cesarean section	0.881 (0.752, 1.032)	0.116	1.243 (1.043, 1.480)	0.015
Vaginal delivery	1.000		1.000	
Feeding patterns within 1 mon (breastfeeding)				
Yes	0.881 (0.715, 1.085)	0.232	0.975 (0.763, 1.245)	0.837
No	1.000		1.000	

^a^infants with GA of 37–38 wk were categorized in early-term, those with GA of 39–41 wk were categorized in full-term. ^b^birth weight was categorized in quartile, Q1 represented those with birth weight of less than 3.08 kg, Q2 represented 3.08–3.34 kg, Q3 represented 3.34–3.60 kg, and Q4 represented greater than 3.60 kg. *P* value < 0.05 represented statistical significance. *GA* gestational age, *BAZ* BMI-for-age Z-score, *OR* odds ratio, *95% CI* 95% confidence interval

**Table 6:** Analysis of prenatal and postnatal factors on BAZ trajectory

Variables	Low trajectory		High trajectory	
	OR (95% CI)	*P* value	OR (95% CI)	*P* value
Prenatal factors				
Parity				
Multipara	1.063 (0.856, 1.320)	0.579	1.265 (1.003, 1.594)	0.047
Primigravida	1.000		1.000	
Gestational hypertension				
Yes	0.703 (0.323, 1.531)	0.375	2.581 (1.387, 4.801)	0.003
No	1.000		1.000	
Postnatal factors				
Birth weight categories^a^				
Q1	1.470 (1.212, 1.782)	< 0.001	0.615 (0.447, 0.847)	0.003
Q2	1.000		1.000	
Q3	0.828 (0.670, 1.023)	0.080	1.440 (1.112, 1.865)	0.006
Q4	0.481 (0.374, 0.617)	< 0.001	2.582 (2.029, 3.287)	< 0.001
Sex				
Boys	0.975 (0.838, 1.135)	0.742	1.314 (1.098, 1.572)	0.003
Girls	1.000		1.000	
Mode of delivery				
Cesarean section	0.953 (0.810, 1.121)	0.559	1.052 (0.877, 1.263)	0.582
Vaginal delivery	1.000		1.000	

^a^birth weight was categorized in quartile, Q1 represented those with birth weight of less than 3.08 kg, Q2 represented 3.08–3.34 kg, Q3 represented 3.34–3.60 kg, and Q4 represented greater than 3.60 kg. *P* value < 0.05 represented statistical significance. *BAZ* BMI-for-age Z-score, *OR* odds ratio, *95% CI* 95% confidence interval

## Discussion

This study identified growth trajectories from 0 to 2 years using both GMM and KML. Subsequently, we compared associations of these methodologically-derived trajectories, and their respective groupings, with BAZ or possible risk of being overweight at three years of age. These analyses revealed distinct growth patterns dependent on methodological approach. The four-category trajectory model derived from KML methodology demonstrated superior performance, with its high trajectory group showing the strongest association with BAZ at three years, and providing superior predictive accuracy for the risk of being overweight at this age. This model was selected as the most appropriate framework for investigating influencing factors in this study. Analyses demonstrated that male sex, high birth weight, multiparity and gestational hypertension were significant risk factors for a high growth trajectory. Of note, high trajectory group was characterized by a rapid and substantial increase in BAZ before six months of age, suggesting rapid weight gain in early infancy. This observation aligns with the literature highlighting the speed of early-life growth as a critical determinant of later health [6, 12].

In major birth cohorts such as Boston Birth Cohort (BBC), Born in Bradford (BiB), INfanciay Medio Ambiente (INMA), Helsinki Birth Cohort Study (HBCS), and Growing Up in Singapore Towards healthy Outcomes (GUSTO) [6, 10, 12, 33, 34], BMI trajectories have been consistently categorized into 3–5 distinct classes. Individuals exhibiting early or late growth acceleration have been associated with a higher risk of being overweight in later life, more severe accumulation of liver and abdominal fat, as well as increased insulin resistance and elevated blood pressure. Collectively this indicated an elevated long-term cardiometabolic disease risk. Furthermore, the HBCS cohort [6] demonstrated that such trajectories can independently predict cancer incidence or all-cause mortality before 70 years of age. The four trajectory groups identified in our study align with the findings from these multiple international cohorts. Of note, the high trajectory group showed superior predictive value for being overweight and obese in later childhood. These findings underscore the significant practical importance of monitoring early-life growth trajectories as a strategic approach for the prevention of being overweight and obese in later childhood.

Two mainstream trajectory modeling methods, GMM and KML, were applied. Consistent with the issues raised by Massara et al. [13] and Lu et al. [14], our results indicated that the growth patterns is highly method-dependent. For instance, although both methods identified a "high trajectory" group, the parametric GMM classified a larger proportion of infants into this group (16.29%) compared to the data-driven KML method (13.61%). However, our study extended previous methodological comparisons by validating these algorithm-derived clusters against a concrete clinical outcome. Despite the theoretical advantages of GMM in handling missing data and providing a probabilistic classification, its high trajectory group demonstrated a significantly weaker association with a risk of being overweight at age three (*β* = 0.440) and a lower predictive accuracy (AUC = 0.571). In contrast, the distribution-free KML method demonstrated superior clinical discrimination (*β* = 1.275; AUC = 0.749). To ensure a thorough comparison, we further performed probability-weighted analyses for the GMM model to account for classification uncertainty. Despite the use of this rigorous methodology, the maximum weighted AUC achieved by the GMM four-class model did not surpass that observed in the KML analysis. For highly variable growth data for infants and toddlers, the data-driven KML method can more accurately identify truly at-risk individuals. These finding has been further validated in the domestic Peking University Birth Cohort in Tongzhou (PKUBC-T) cohort [35].

Comprehensive analysis indicates that distinct developmental trajectories of childhood obesity are associated with specific risk factor profiles. While Pryor et al. [21] identified maternal overweight or obesity categorization as the strongest driver of offspring obesity trajectories and reported no statistically significant association between birth weight and BMI trajectories of children, our study found high birth weight is a risk factor for high BMI trajectories. Similarly, Begum et al. [36] reported that gestational hypertension, high birth weight, and cesarean delivery significantly increased the risk of accelerated BMI trajectories in childhood. Meng et al. [37] also confirmed that male sex is a risk factor for accelerated BMI growth trajectories during infancy and toddlerhood, whereas primiparity is associated with a lower risk of such acceleration. Thus, infants born to multiparous mothers appear to be at greater risk for accelerated growth. These risk factors may operate through distinct biological mechanisms. Male sex primarily drives accelerated weight gain during infancy and toddlerhood via early-life hormonal differences, such as testosterone [38]. High birth weight, largely resulting from intrauterine nutrient surplus, establishes a postnatal pattern of sustained rapid growth through mechanisms involving placental function and epigenetic programming. Multiparity may be associated with adaptive changes in placental function, leading to higher birth weight and subsequently influencing growth velocity during infancy [39]. Gestational hypertension, alters the maternal-placental metabolic milieu and triggers fetal intrauterine stress and adaptive responses, thereby programming fetal metabolism and elevating the risk of future high BMI trajectories [40].

A key strength of this study is the application and comparison of both GMM and KML methods, providing the robust evidence to inform the selection of appropriate trajectory modeling approaches. We also conducted a comprehensive analysis to clarify the associations between multiple prenatal and postnatal factors and specific growth patterns. Our findings offer important insights into early-life growth patterns and pinpoint significant antenatal and postnatal factors associated with high BAZ trajectories. Managing gestational hypertension, and maintaining optimal birth weight may help mitigate this early rapid BAZ gain. The non-modifiable risk factors found in our study can guide the development of targeted early surveillance programs and personalized preventive strategies. Furthermore, using the longitudinal data mitigates limitations inherent in cross-sectional studies and enhances the validity of research examining the relationship between early BAZ trajectories and later health outcomes. Future research could explore more advanced machine learning techniques to further optimize predictive accuracy for early childhood overweight risks.

This study still had several limitations. First, all participants were recruited from a single medical center, which may limit generalizability. Future studies with larger, multi-center samples are needed to validate these findings. Second, only 1464 had BAZ measurements available at three years of age for outcome prediction. This reduction in sample size at later follow-up points could introduce selection bias. Therefore, we conducted a sensitivity analysis comparing the baseline characteristics of children with and without 3-year BAZ measurements (Supplementary Table 6). This analysis revealed no significant differences for most key demographic and maternal characteristics. Future studies will adopt strategies to minimize attrition and further investigate the specific impact of such biases. Third, the follow-up period of this study was relatively short, extending to only three years of age. While this period is critical for early life interventions, it restricts the ability to assess the long-term predictive efficacy of these BAZ trajectories for being overweight or obese in childhood or adolescence. Future research could aim to conduct extended follow-up to verify these longer-term outcomes.

In conclusion, this study used GMM and KML to analyze BAZ, finding that a KML-derived four-trajectory model demonstrated superior performance in predicting age three risk of being overweight. Analyses based on this model identified key antenatal and postnatal factors, including male sex, high birth weight, multiparity and gestational hypertension, as significant contributors to high BAZ trajectories. This study establishes a robust methodological framework for assessing early-life BMI development and underscore the profound impact of prenatal and early postnatal determinants. These findings provide valuable insights for the primary prevention of childhood obesity. Future research should investigate the complex interactions among these risk factors to inform targeted interventions.

## Supplementary Information

Below is the link to the electronic supplementary material.

Supplementary file1 (DOCX 769 KB)

## Data Availability

Data from this study will be available by contacting the corresponding authors. Ethics consent will be required.

## References

[1] Mameli C, Mazzantini S, Zuccotti GV. Nutrition in the first 1000 days: the origin of childhood obesity. Int J Environ Res Public Health. 2016;13:838. 10.3390/ijerph13090838. 27563917 10.3390/ijerph13090838 PMC5036671

[2] Heiskala A, Tucker JD, Choudhary P, Nedelec R, Ronkainen J, Sarala O, et al. Timing based clustering of childhood Bmi trajectories reveals differential maturational patterns; study in the Northern Finland Birth Cohorts 1966 and 1986. Int J Obes (Lond). 2025;49:872–80. 10.1038/s41366-025-01714-8. 39820013 10.1038/s41366-025-01714-8 PMC12095082

[3] Tung JYL, Poon GWK, Du J, Wong KKY. Obesity in children and adolescents: overview of the diagnosis and management. Chronic Dis Transl Med. 2023;9:122–33. 10.1002/cdt3.58. 37305109 10.1002/cdt3.58 PMC10249183

[4] World Health Organization, United Nations Children's Fund (UNICEF), World Bank Group. Levels and Trends in Child Malnutrition: UNICEF /WHO/World Bank Group Joint Child Malnutrition Estimates: Key Findings of the 2025 Edition. Geneva: World Health Organization; 2025. Available from: https://iris.who.int/handle/10665/381846. Accessed 6 Jan 2026.

[5] The State Council Information Office of the People's Republic of China (SCIO). Scio Holds News Conference on High-Quality Implementation of 14th Five-Year Plan, Highlighting Health Achievements. Available from: http://www.scio.gov.cn/live/2025/37226/index.html. Accessed 6 Jan 2026.

[6] von Bonsdorff MB, Törmäkangas T, Rantanen T, Salonen MK, Osmond C, Kajantie E, et al. Early life body mass trajectories and mortality in older age: findings from the Helsinki Birth Cohort Study. Ann Med. 2015;47:34–9. 10.3109/07853890.2014.963664. 25307361 10.3109/07853890.2014.963664

[7] Ames ME, Wintre MG. Growth mixture modeling of adolescent body mass index development: longitudinal patterns of internalizing symptoms and physical activity. J Res Adolesc. 2016;26:889–901. 10.1111/jora.12239. 28453209 10.1111/jora.12239

[8] Wu YF, Fan HY, Chen YC, Kuo KL, Chien KL. Adolescent tri-ponderal mass index growth trajectories and incident diabetes mellitus in early adulthood. J Clin Endocrinol Metab. 2021;106:e2919–27. 10.1210/clinem/dgab235. 33839769 10.1210/clinem/dgab235

[9] Movin M, Garden FL, Protudjer JL, Ullemar V, Svensdotter F, Andersson D, et al. Impact of childhood asthma on growth trajectories in early adolescence: findings from the Childhood Asthma Prevention Study (Caps). Respirology. 2017;22:460–5. 10.1111/resp.12928. 27859946 10.1111/resp.12928

[10] Mebrahtu TF, Feltbower RG, Petherick ES, Parslow RC. Growth patterns of White British and Pakistani children in the Born in Bradford Cohort: a latent growth modelling approach. J Epidemiol Community Health. 2015;69:368–73. 10.1136/jech-2014-204571. 25492896 10.1136/jech-2014-204571

[11] Genolini C, Falissard B. Kml: a package to cluster longitudinal data. Comput Methods Programs Biomed. 2011;104:e112–21. 10.1016/j.cmpb.2011.05.008. 21708413 10.1016/j.cmpb.2011.05.008

[12] Huang W, Meir AY, Olapeju B, Wang G, Hong X, Venkataramani M, et al. Defining longitudinal trajectory of body mass index percentile and predicting childhood obesity: methodologies and findings in the boston birth cohort. Precis Nutr. 2023;2:e00037. 10.1097/pn9.0000000000000037. 37745028 10.1097/PN9.0000000000000037 PMC10513013

[13] Massara P, Keown-Stoneman CD, Erdman L, Ohuma EO, Bourdon C, Maguire JL, et al. Identifying longitudinal-growth patterns from infancy to childhood: a study comparing multiple clustering techniques. Int J Epidemiol. 2021;50:1000–10. 10.1093/ije/dyab021. 33693803 10.1093/ije/dyab021

[14] Lu Z, Ahmadiankalati M, Tan Z. Joint clustering multiple longitudinal features: a comparison of methods and software packages with practical guidance. Stat Med. 2023;42:5513–40. 10.1002/sim.9917. 37789706 10.1002/sim.9917

[15] Mattsson M, Maher GM, Boland F, Fitzgerald AP, Murray DM, Biesma R. Group-based trajectory modelling for Bmi trajectories in childhood: a systematic review. Obes Rev. 2019;20:998–1015. 10.1111/obr.12842. 30942535 10.1111/obr.12842

[16] van Biljon N, Lake MT, Goddard L, Botha M, Zar HJ, Little F. Latent classes of anthropometric growth in early childhood using uni- and multivariate approaches in a South African birth cohort. PLoS ONE. 2025;20:e0319237. 10.1371/journal.pone.0319237. 40131891 10.1371/journal.pone.0319237 PMC11936193

[17] Stuart B, Panico L. Early-childhood Bmi trajectories: evidence from a prospective, nationally representative British cohort study. Nutr Diabetes. 2016;6:e198. 10.1038/nutd.2016.6. 26950479 10.1038/nutd.2016.6 PMC4817077

[18] Tu AW, Masse LC, Lear SA, Gotay CC, Richardson CG. Body mass index trajectories from ages 1 to 20: results from two nationally representative Canadian longitudinal cohorts. Obesity. 2015;23:1703–11. 10.1002/oby.21158. 26179716 10.1002/oby.21158

[19] Ziyab AH, Karmaus W, Kurukulaaratchy RJ, Zhang H, Arshad SH. Developmental trajectories of body mass index from infancy to 18 years of age: prenatal determinants and health consequences. J Epidemiol Community Health. 2014;68:934–41. 10.1136/jech-2014-203808. 24895184 10.1136/jech-2014-203808 PMC4174013

[20] Magee CA, Caputi P, Iverson DC. Identification of distinct body mass index trajectories in Australian children. Pediatr Obes. 2013;8:189–98. 10.1111/j.2047-6310.2012.00112.x. 23143781 10.1111/j.2047-6310.2012.00112.x

[21] Pryor LE, Tremblay RE, Boivin M, Touchette E, Dubois L, Genolini C. Developmental trajectories of body mass index in early childhood and their risk factors: an 8-year longitudinal study. Arch Pediatr Adolesc Med. 2011;165:906–12. 10.1001/archpediatrics.2011.153. 21969392 10.1001/archpediatrics.2011.153

[22] Ventura AK, Loken E, Birch LL. Developmental trajectories of girls’ Bmi across childhood and adolescence. Obesity. 2009;17:2067–74. 10.1038/oby.2009.123. 19424165 10.1038/oby.2009.123 PMC12908222

[23] Aris IM, Chen LW, Tint MT, Pang WW, Soh SE, Saw SM, et al. Body Mass Index trajectories in the first two years and subsequent childhood cardio-metabolic outcomes: a prospective multi-ethnic Asian cohort study. Sci Rep. 2017;7:8424. 10.1038/s41598-017-09046-y. 28827610 10.1038/s41598-017-09046-y PMC5567284

[24] Woo Baidal JA, Locks LM, Cheng ER, Blake-Lamb TL, Perkins ME, Taveras EM. Risk factors for childhood obesity in the first 1,000 days: a systematic review. Am J Prev Med. 2016;50:761–79. 10.1016/j.amepre.2015.11.012. 26916261 10.1016/j.amepre.2015.11.012

[25] Li X, Keown-Stoneman CDG, Lebovic G, Omand JA, Adeli K, Hamilton JK, et al. The association between Body Mass Index trajectories and cardiometabolic risk in young children. Pediatr Obes. 2020;15:e12633. 10.1111/ijpo.12633. 32181602 10.1111/ijpo.12633

[26] Goulet D, Boivin M, Gravel CA, Little J, Potter BK, Dubois L. Mediation of genetic susceptibility to obesity through eating behaviours in children. Pediatr Obes. 2025;20:e13180. 10.1111/ijpo.13180. 39390328 10.1111/ijpo.13180 PMC11936709

[27] WHOMGRS. Who child growth standards based on length/height, weight and age. Acta Paediatr Suppl. 2006;450:76–85. 10.1111/j.1651-2227.2006.tb02378.x. 16817681 10.1111/j.1651-2227.2006.tb02378.x

[28] de Onis M, Lobstein T. Defining obesity risk status in the general childhood population: which cut-offs should we use? Int J Pediatr Obes. 2010;5:458–60. 10.3109/17477161003615583. 20233144 10.3109/17477161003615583

[29] Bravo MA, Zephyr D, Fiffer MR, Miranda ML. Weekly prenatal Pm(2.5) and No(2) exposures in preterm, early term, and full term infants: decrements in birth weight and critical windows of susceptibility. Environ Res. 2024;240:117509. 10.1016/j.envres.2023.117509. 37890819 10.1016/j.envres.2023.117509 PMC10842146

[30] Attali E, Yogev Y. The impact of advanced maternal age on pregnancy outcome. Best Pract Res Clin Obstet Gynaecol. 2021;70:2–9. 10.1016/j.bpobgyn.2020.06.006. 32773291 10.1016/j.bpobgyn.2020.06.006

[31] Nguena Nguefack HL, Page MG, Katz J, Choiniere M, Vanasse A, Dorais M, et al. Trajectory modelling techniques useful to epidemiological research: a comparative narrative review of approaches. Clin Epidemiol. 2020;12:1205–22. 10.2147/CLEP.S265287. 33154677 10.2147/CLEP.S265287 PMC7608582

[32] Berlin KS, Parra GR, Williams NA. An introduction to latent variable mixture modeling (Part 2): longitudinal latent class growth analysis and growth mixture models. J Pediatr Psychol. 2014;39:188–203. 10.1093/jpepsy/jst085. 24277770 10.1093/jpepsy/jst085

[33] Montazeri P, Vrijheid M, Martinez D, Basterrechea M, Fernandez-Somoano A, Guxens M, et al. Maternal metabolic health parameters during pregnancy in relation to early childhood BMI trajectories. Obesity. 2018;26:588–96. 10.1002/oby.22095. 29399981 10.1002/oby.22095

[34] Michael N, Gupta V, Fogel A, Huang J, Chen L, Sadananthan SA, et al. Longitudinal characterization of determinants associated with obesogenic growth patterns in early childhood. Int J Epidemiol. 2023;52:426–39. 10.1093/ije/dyac177. 36087338 10.1093/ije/dyac177 PMC10114026

[35] Wang SS, Yue ZH, Han N, Lyu JL, Ji YL, Wang H, et al. Association of Maternal Pre-Pregnancy Bmi, Gestational Weight Gain, and Gestational Diabetes Mellitus with Bmi Trajectory in Early Childhood: A Prospective Cohort Study. Zhonghua Liu Xing Bing Xue Za Zhi. 2024;45:1348–55. 10.3760/cma.j.cn112338-20240513-00270. 39444117 10.3760/cma.j.cn112338-20240513-00270

[36] Begum T, Fatima Y, Perales F, Anuradha S, Mamun A. Associations of caesarean section with body mass and waist circumference trajectories from age 2 to 13 years: a nationally representative birth cohort study in Australia. Pediatr Obes. 2021;16:e12769. 10.1111/ijpo.12769. 33403832 10.1111/ijpo.12769

[37] Meng X, Wang J, Zhang N, Li X, Wu Q. Application of group-based trajectory models to evaluate the association of fetal growth trajectories and childhood overweight and obesity: a longitudinal study with 2-year follow-up. PLoS ONE. 2025;20:e0330715. 10.1371/journal.pone.0330715. 40961025 10.1371/journal.pone.0330715 PMC12443285

[38] Lucaccioni L, Trevisani V, Boncompagni A, Marrozzini L, Berardi A, Iughetti L. Minipuberty: looking back to understand moving forward. Front Pediatr. 2020;8:612235. 10.3389/fped.2020.612235. 33537266 10.3389/fped.2020.612235 PMC7848193

[39] Hinkle SN, Albert PS, Mendola P, Sjaarda LA, Yeung E, Boghossian NS, et al. The association between parity and birthweight in a longitudinal consecutive pregnancy cohort. Paediatr Perinat Epidemiol. 2014;28:106–15. 10.1111/ppe.12099. 24320682 10.1111/ppe.12099 PMC3922415

[40] Yan S, Lyu J, Liu Z, Zhou S, Ji Y, Wang H. Association of gestational hypertension and preeclampsia with offspring adiposity: a systematic review and meta-analysis. Front Endocrinol (Lausanne). 2022;13:906781. 10.3389/fendo.2022.906781. 36082079 10.3389/fendo.2022.906781 PMC9445980

