# Supplementary material for: BMI-for-age Z-score trajectories in early childhood: a comparison of GMM and KML and associated antenatal and postnatal factors
Source: World J Pediatr. 2026 Jul 23;22(6):664–77. doi: 10.1007/s12519-026-01057-y (PMC13433460; doi:10.1007/s12519-026-01057-y)
Supplement: Supplementary file 1 [file 12519_2026_1057_MOESM1_ESM.docx]

**Supplementary Information**

**Detailed specification of growth mixture modeling (GMM)**

Latent class trajectory analysis based on GMM was carried out using “lcmm” package [1]. The fixed effect term of the model included the natural cubic spline function of age and sex, and the random effect term included the random intercept and the random slope of age. Although BMI-for-Age *Z*-score (BAZ) scores are standardized for age and sex at each specific time point, age and sex are still included in GMM allows the identification of age-dependent trajectory patterns and sex-specific differences in the evolution of BAZ over time. This approach helps capture subgroups with different dynamic growth trends that may otherwise be obscured by cross-sectional standardization alone. The optimal number of latent trajectory groups was determined by balancing statistical fit, model parsimony, and clinical interpretability.

Reference criteria included minimization of Bayesian information criterion (BIC), maximization of entropy, average of posterior probability of assignment (APPA) for each group > 70% and the proportion of the minimum group > 5%. We conducted probability-weighted logistic regression analyses for the predictive performance of the GMM model. In these analyses, each individual's posterior probability of belonging to their most likely latent class was used as a sampling weight. The weighted area under the curve (AUC) and its 95% confidence interval (95%CI) were calculated for crude and adjusted models.

**Detailed specification of k-means for longitudinal data (KML)**

To ensure methodological transparency, the specific configurations for the KML algorithm were as follows: Euclidean distance was used as the dissimilarity measure to cluster the longitudinal trajectories. To avoid the algorithm converging on local minima, the pre-configuration included 100 iterations/restarts (nbRedrawing = 100) for each specified number of clusters (k = 2 to 5). A random seed (set.seed (101)) was set to ensure reproducibility. Intermittent missing values in the longitudinal data were automatically handled by a built-in imputation function in the package prior to the clustering step. Cluster validation and determination of the optimal number of trajectory groups were based on identifying the peak of the Calinski-Harabasz index, supplemented by clinical interpretability and ensuring no cluster contained fewer than 5% of the total sample.

References

1. Nguena Nguefack HL, Page MG, Katz J, Choiniere M, Vanasse A, Dorais M, et al. Trajectory Modelling Techniques Useful to Epidemiological Research: A Comparative Narrative Review of Approaches. Clin Epidemiol. 2020;12:1205-22. doi: 10.2147/CLEP.S265287.

| **Supplementary Table 1** Distribution of data included in trajectory analysis | | | | | |
| --- | --- | --- | --- | --- | --- |
| Time point (mon) | Number | Age (mon) | BAZ | BAZ > 1 | BAZ > 2 |
| 0 | 4629 | 0 ± 0 | -0.15 ± 0.91 | 462 (9.98) | 30 (0.65) |
| 1 | 3825 | 1.4 ± 0.1 | -0.14 ± 0.85 | 327 (8.55) | 17 (0.44) |
| 3 | 3772 | 3.2 ± 0.4 | 0.19 ± 0.98 | 787 (20.86) | 126 (3.34) |
| 6 | 4092 | 6.2 ± 0.4 | 0.21 ± 1.03 | 883 (21.58) | 179 (4.37) |
| 9 | 3479 | 8.9 ± 0.8 | 0.11 ± 0.98 | 618 (17.76) | 94 (2.70) |
| 12 | 3749 | 12.3 ± 0.6 | 0.03 ± 0.93 | 539 (14.38) | 71 (1.89) |
| 18 | 2847 | 18.4 ± 0.6 | -0.19 ± 0.91 | 262 (9.20) | 31 (1.09) |
| 24 | 3525 | 24.4 ± 0.6 | -0.52 ± 0.95 | 199 (5.65) | 22 (0.62) |

Data are presented as mean ± standard deviation (SD) or *n* (%). *BAZ* BMI-for-age Z-score.

| **Supplementary Table 2** BAZ at different time points for the four-class trajectories determined using the GMM method | | | | | | | | | | |
| --- | --- | --- | --- | --- | --- | --- | --- | --- | --- | --- |
| Time point (mon) | Number | BAZ  mean (SD) | | | |  | BAZ > 1 number (%) | | | |
|  |  | Class 1 (*N* = 1057) | Class 2 (*N* = 2063) | Class 3 (*N* = 755) | Class 4 (*N* = 754) |  | Class 1 (*N* = 1057) | Class 2 (*N* = 2063) | Class 3 (*N* = 755) | Class 4 (*N* = 754) |
| 0 | 4629 | 0.42 (0.79) | -0.20 (0.78) | -0.10 (0.84) | -0.85 (0.94) |  | 236 (22.33) | 141 (6.83) | 70 (9.27) | 15 (1.99) |
| 1 | 3825 | -0.39 (0.73) | 0.10 (0.74) | -0.71 (0.83) | 0.13 (0.89) |  | 24 (2.27) | 187 (9.06) | 11 (1.46) | 105 (13.93) |
| 3 | 3772 | -0.65 (0.77) | 0.40 (0.77) | -0.16 (0.82) | 1.10 (0.85) |  | 19 (1.80) | 371 (17.98) | 46 (6.09) | 351 (46.55) |
| 6 | 4092 | -0.77 (0.75) | 0.19 (0.76) | 0.54 (0.81) | 1.30 (0.91) |  | 9 (0.85) | 273 (13.23) | 181 (23.97) | 420 (55.70) |
| 9 | 3479 | -0.75 (0.76) | 0.01 (0.72) | 0.65 (0.77) | 1.04 (0.89) |  | 6 (0.57) | 128 (6.20) | 180 (23.84) | 304 (40.32) |
| 12 | 3749 | -0.71 (0.76) | -0.13 (0.71) | 0.60 (0.79) | 0.85 (0.81) |  | 7 (0.66) | 85 (4.12) | 190 (25.17) | 257 (34.08) |
| 18 | 2847 | -0.78 (0.81) | -0.30 (0.76) | 0.36 (0.84) | 0.39 (0.82) |  | 8 (0.76) | 59 (2.86) | 96 (17.72) | 99 (13.13) |
| 24 | 3525 | -0.96 (0.89) | -0.63 (0.86) | -0.03 (0.94) | -0.10 (0.89) |  | 13 (1.23) | 45 (2.18) | 74 (9.80) | 67 (8.89) |

*BAZ* BMI-for-age *Z*-score; *GMM* growth mixture modeling; *SD* standard deviation.

| **Supplementary Table 3** BAZ at different time points for the two-class trajectories using the KML method | | | | | | |
| --- | --- | --- | --- | --- | --- | --- |
| Time point (mon) | Number | BAZ  mean (SD) | |  | BAZ > 1 number (%) | |
|  |  | Class 1 (*N* = 2413) | Class 2 (*N* = 2216) |  | Class 1 (*N* = 2413) | Class 2 (*N* = 2216) |
| 0 | 4629 | -0.42 (0.87) | 0.15 (0.87) |  | 101 (4.19) | 361 (16.29) |
| 1 | 3825 | -0.56 (0.72) | 0.32 (0.72) |  | 17 (0.70) | 310 (13.99) |
| 3 | 3772 | -0.45 (0.71) | 0.88 (0.73) |  | 29 (1.20) | 758 (34.21) |
| 6 | 4092 | -0.50 (0.69) | 0.98 (0.74) |  | 9 (0.37) | 874 (39.44) |
| 9 | 3479 | -0.57 (0.66) | 0.84 (0.69) |  | 3 (0.12) | 615 (27.75) |
| 12 | 3749 | -0.61 (0.65) | 0.70 (0.68) |  | 9 (0.37) | 530 (23.92) |
| 18 | 2847 | -0.77 (0.66) | 0.44 (0.69) |  | 2 (0.08) | 260 (11.73) |
| 24 | 3525 | -1.11 (0.70) | 0.12 (0.75) |  | 4 (0.17) | 195 (8.8) |

*BAZ* BMI-for-age *Z*-score; *KML* k-means for longitudinal data; *SD* standard deviation.

| **Supplementary Table 4** BAZ at different time points for the three-class trajectories determined using the KML method | | | | | | | | |
| --- | --- | --- | --- | --- | --- | --- | --- | --- |
| Time point (mon) | Number | BAZ  mean (SD) | | |  | BAZ > 1 number (%) | | |
|  |  | Class 1 (*N* = 1345) | Class 2 (*N* = 2071) | Class 3 (*N* = 1213) |  | Class 1 (*N* = 1345) | Class 2 (*N* = 2071) | class3 (*N* = 1213) |
| 0 | 4629 | -0.52 (0.88) | -0.14 (0.84) | 0.26 (0.88) |  | 51 (3.79) | 166 (8.02) | 245 (20.20) |
| 1 | 3825 | -0.80 (0.68) | -0.12 (0.66) | 0.54 (0.72) |  | 3 (0.22) | 61 (2.95) | 263 (21.68) |
| 3 | 3772 | -0.79 (0.63) | 0.22 (0.60) | 1.22 (0.68) |  | 1 (0.07) | 168 (8.11) | 618 (50.95) |
| 6 | 4092 | -0.87 (0.62) | 0.22 (0.53) | 1.39 (0.67) |  | 1 (0.07) | 126 (6.08) | 756 (62.32) |
| 9 | 3479 | -0.94 (0.59) | 0.12 (0.48) | 1.24 (0.63) |  | 1 (0.07) | 57 (2.75) | 560 (46.17) |
| 12 | 3749 | -0.95 (0.57) | 0.03 (0.50) | 1.07 (0.62) |  | 1 (0.07) | 41 (1.98) | 497 (40.97) |
| 18 | 2847 | -1.09 (0.62) | -0.17 (0.53) | 0.78 (0.66) |  | 0 (0.00) | 17 (0.82) | 245 (20.20) |
| 24 | 3525 | -1.42 (0.65) | -0.52 (0.60) | 0.47 (0.71) |  | 1 (0.07) | 11 (0.53) | 187 (15.42) |

*BAZ* BMI-for-age Z-score; *KML* k-means for longitudinal data; *SD* standard deviation.

| **Supplementary Table 5** BAZ at different time points for the four-class trajectories determined using the KML method | | | | | | | | | | |
| --- | --- | --- | --- | --- | --- | --- | --- | --- | --- | --- |
| Time point (mon) | Number | BAZ  mean (SD) | | | |  | BAZ > 1 number (%) | | | |
|  |  | Class 1 (*N* = 886) | Class 2 (*N* = 1598) | Class 3 (*N* = 1515) | Class 4 (*N* = 630) |  | Class 1 (*N* = 886) | Class 2 (*N* = 1598) | Class 3 (*N* = 1515) | Class 4 (*N* = 630) |
| 0 | 4629 | -0.54 (0.90) | -0.41 (0.83) | 0.16 (0.82) | 0.33 (0.87) |  | 32 (3.61) | 58 (3.63) | 233 (15.38) | 139 (22.06) |
| 1 | 3825 | -0.89 (0.68) | -0.39 (0.68) | 0.20 (0.64) | 0.71 (0.73) |  | 2 (0.23) | 14 (0.88) | 126 (8.32) | 185 (29.37) |
| 3 | 3772 | -0.96 (0.60) | -0.14 (0.58) | 0.67 (0.58) | 1.48 (0.69) |  | 0 (0.00) | 31 (1.94) | 351 (23.17) | 405 (64.29) |
| 6 | 4092 | -1.10 (0.56) | -0.13 (0.51) | 0.71 (0.52) | 1.72 (0.68) |  | 0 (0.00) | 15 (0.94) | 392 (25.87) | 476 (75.56) |
| 9 | 3479 | -1.16 (0.55) | -0.20 (0.47) | 0.56 (0.50) | 1.56 (0.62) |  | 0 (0.00) | 10 (0.63) | 216 (14.26) | 392 (62.22) |
| 12 | 3749 | -1.14 (0.54) | -0.25 (0.51) | 0.42 (0.51) | 1.38 (0.61) |  | 0 (0.00) | 17 (1.06) | 145 (9.57) | 377 (59.84) |
| 18 | 2847 | -1.30 (0.59) | -0.43 (0.52) | 0.14 (0.52) | 1.12 (0.61) |  | 0 (0.00) | 5 (0.31) | 38 (2.51) | 219 (34.76) |
| 24 | 3525 | -1.59 (0.63) | -0.79 (0.60) | -0.15 (0.61) | 0.76 (0.70) |  | 1 (0.11) | 4 (0.25) | 31 (2.05) | 163 (25.87) |

*BAZ* BMI-for-age Z-score; *KML* k-means for longitudinal data; *SD* standard deviation.

| **Supplementary Table 6** Baseline characteristics of children with and without BAZ measurements at 3 y of age | | | |
| --- | --- | --- | --- |
| **Characteristics** | **without 3 y BAZ data (*N* = 3165)** | **with 3 y BAZ data (*N* = 1464)** | ***P* value** |
| **Infants** |  |  |  |
| Sex |  |  | 0.377 |
| boy | 1653 (52.2%) | 785 (53.6%) |  |
| girl | 1512 (47.8%) | 679 (46.4%) |  |
| Mode of delivery |  |  | 0.579 |
| Vaginal delivery | 2035 (64.3%) | 929 (63.5%) |  |
| Cesarean section | 1130 (35.7%) | 535 (36.5%) |  |
| Categorized by GA ^a^ |  |  | 0.162 |
| Early-term | 875 (27.6%) | 376 (25.7%) |  |
| Full-term | 2290 (72.4%) | 1088 (74.3%) |  |
| Categorized by birth weight ^b^ |  |  | 0.089 |
| Q1 | 823 (26.0%) | 341 (23.3%) |  |
| Q2 | 815 (25.8%) | 361 (24.7%) |  |
| Q3 | 774 (24.5%) | 379 (25.9%) |  |
| Q4 | 753 (23.8%) | 383 (26.2%) |  |
| Feeding patterns within 1 mon (breastfeeding) | 2721 (86.0%) | 1232 (84.3%) | 0.140 |
| **Mothers** |  |  |  |
| Advanced maternal age |  |  | 0.003 |
| Yes | 209 (6.6%) | 133 (9.1%) |  |
| No | 2956 (93.4%) | 1331 (90.9%) |  |
| Parity |  |  | 0.065 |
| Primigravida | 2705 (85.5%) | 1218 (83.4%) |  |
| Multipara | 460 (14.5%) | 243 (16.6%) |  |
| Threatened miscarriage |  |  | 0.077 |
| Yes | 36 (1.1%) | 26 (1.8%) |  |
| No | 3129 (98.9%) | 1435 (98.2%) |  |
| Infections in pregnancy |  |  | 0.319 |
| Yes | 29 (0.9%) | 18 (1.2%) |  |
| No | 3136 (99.1%) | 1443 (98.8%) |  |
| Gestational hypertension |  |  | 0.166 |
| Yes | 46 (1.5%) | 14 (1.0%) |  |
| No | 3119 (98.5%) | 1447 (99.0%) |  |
| Gestational diabetes mellitus |  |  | 0.317 |
| Yes | 81 (2.6%) | 45 (3.1%) |  |
| No | 3084 (97.4%) | 1419(96.9%) |  |
| Thyroid dysfunction in pregnancy |  |  | 0.700 |
| Yes | 72 (2.3%) | 36 (2.5%) |  |
| No | 3093 (97.7%) | 1428 (97.5%) |  |

^a^ infants with GA of 37–38 wk were categorized in early-term, those with GA of 39–41 wk were categorized in full-term. ^b^ birth weight was categorized in quartile, Q1 represented those with birth weight of less than 3.08 kg, Q2 represented 3.08–3.34 kg, Q3 represented 3.34–3.60 kg, and Q4 represented greater than 3.60 kg. *P* value < 0.05 represented statistical significance. *GA* gestational age; *BAZ* BMI-for-age Z-score.

Supplementary Fig. 1 Comparison of Calinski-Harabasz indices across different cluster numbers, iteration counts, and sexes. **a** results of boys; **b** results of girls.

Supplementary Fig. 2 BAZ trajectories in children identified by the two-class and three-class KML methods. *BAZ* BMI-for-age *Z*-score; *mon* month.
